# Assessment of CareStart G6PD rapid diagnostic test and CareStart G6PD biosensor in Mauritania

**DOI:** 10.1186/s40249-021-00889-2

**Published:** 2021-08-05

**Authors:** Oum Kelthoum Mamadou Djigo, Yacoub Ould Khalef, Mohamed Salem Ould Ahmedou Salem, Nicolas Gomez, Leonardo Basco, Sébastien Briolant, Ali Ould Mohamed Salem Boukhary

**Affiliations:** 1 Unité de Recherche “Génomes et Milieux” (Jeune Equipe Associée à l’Institut de Recherche pour le Développement), Faculté des Sciences et Techniques, Université de Nouakchott Al-Aasriya, Nouakchott, Mauritania; 2 Service de Pédiatrie, Centre Hospitalier Mère et Enfant, Nouakchott, Mauritania; 3 IHU, Méditerranée Infection, Marseille, France; 4 Aix Marseille Univ, IRD, AP-HM, SSA, VITROME, Marseille, France; 5 Unité de Parasitologie Entomologie, Département de Microbiologie et Maladies Infectieuses, Institut de Recherche Biomédicale des Armées (IRBA), Marseille, France

**Keywords:** Glucose-6-phosphate dehydrogenase, Malaria, *Plasmodium vivax*, Primaquine, Tafenoquine

## Abstract

**Background:**

The elimination of *Plasmodium vivax* malaria requires 8-aminoquinolines, which are contraindicated in patients with glucose-6-phosphate dehydrogenase (G6PD) deficiency due to the risk of acute haemolytic anaemia. Several point-of-care devices have been developed to detect G6PD deficiency. The objective of the present study was to evaluate the performance of two of these devices against G6PD genotypes in Mauritania.

**Methods:**

Outpatients were screened for G6PD deficiency using CareStart™ rapid diagnostic test (RDT) and CareStart™ G6PD biosensor in Nouakchott, Mauritania, in 2019–2020. African-type and Mediterranean-type G6PD genotypes commonly observed in Africa were determined by polymerase chain reaction-restriction fragment length polymorphism and sequencing. Qualitative variables were compared using Fisher’s exact test.

**Results:**

Of 323 patients (74 males and 249 females), 5 males and 2 homozygous females had the African-type A- genotype: A^−(202)^ in 3 males and 2 females and G6PD A^−(968)^ in 2 males. Among heterozygous females, 13 carried G6PD A^−(202)^, 12 G6PD A^−(968)^, and 3 G6PD A^−(542)^ variants. None had the Mediterranean-type G6PD genotype. Eight had a positive G6PD RDT result, including all 7 hemizygous males and homozygous females with A- or A-A- (0.12 to 2.34 IU/g haemoglobin, according to G6PD biosensor), but RDT performed poorly (sensitivity, 11.1% at the cut-off level of < 30%) and yielded many false negative tests. Thirty-seven (50.0%) males and 141 (56.6%) females were anaemic. The adjusted median values of G6PD activity were 5.72 and 5.34 IU/g haemoglobin in non-anaemic males (*n* = 35) and non-anaemic males and females (*n* = 130) with normal G6PD genotypes using G6PD biosensor, respectively. Based on the adjusted median of 5.34 IU/g haemoglobin, the performance of G6PD biosensor against genotyping was as follows: at 30% cut-off, the sensitivity and specificity were 85.7% and 91.7%, respectively, and at 80% cut-off, the sensitivity was 100% while the specificity was 64.9%.

**Conclusions:**

Although this pilot study supports the utility of biosensor to screen for G6PD deficiency in patients, further investigation in parallel with spectrophotometry is required to promote and validate a more extensive use of this point-of-care device in areas where *P. vivax* is highly prevalent in Mauritania.

**Graphic abstract:**

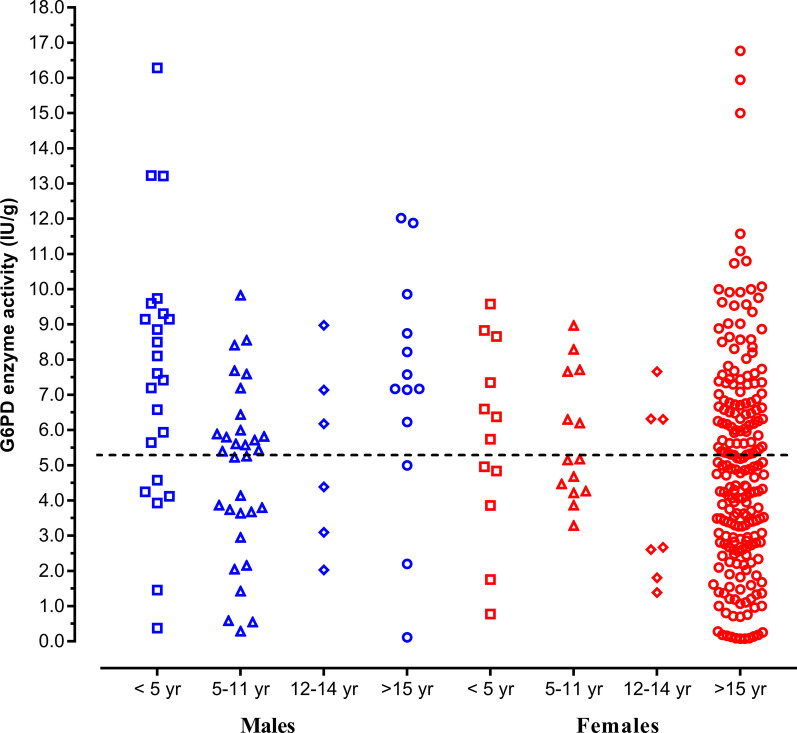

## Background

Glucose-6-phosphate dehydrogenase (G6PD) is the key enzyme of the oxidative phosphogluconate pathway, the only source of nicotinamide adenine dinucleotide phosphate hydrogen (NADPH) in mature erythrocytes, required to generate glutathione and protect erythrocytes from oxidative damage. Human G6PD is encoded by a housekeeping X-linked gene spanning 18.5 kilobases, which is composed of 13 exons and 12 introns [1, 2]. The G6PD gene has undergone numerous mutations, leading to more than 200 G6PD variants with a wide range of enzyme activity [3–5]. Some variants lead to clinical signs and symptoms, but the majority remain clinically silent, and blood profiles are normal. However, when G6PD-deficient individuals are exposed to exogenous triggering factors (drugs, fava beans, infections), low levels of intraerythrocytic NADPH and rapid depletion of glutathione may result in accumulation of reactive oxygen species and destruction of erythrocytes (haemolysis), haemoglobinuria, and hyperbilirubinaemia [6].

G6PD deficiency occurs frequently in human populations residing in malaria endemic areas around the world [7]. Acute haemolytic anaemia has been reported in G6PD-deficient individuals after administration of certain antimalarial drugs, including dapsone, methylene blue, and 8-aminoquinolines (primaquine and tafenoquine) [8–14]. The degree of dapsone- and primaquine-induced acute haemolytic anaemia is dose-dependent [9, 15]. Despite this well-known, potentially fatal adverse effect, 8-aminoquinolines are indispensable to achieve elimination of both *Plasmodium falciparum* and *Plasmodium vivax* [16]. The 8-aminoquinolines have distinct roles for each of these *Plasmodium* species. Mature *P. falciparum* gametocytes survive exposure to blood schizonticides but can be killed with a single low-dose primaquine, which can be administered without prior testing of G6PD status [17–19]. The complete treatment of *P. vivax* malaria, referred to as “radical treatment,” consists of administering at least two drugs targeted against different asexual developmental stages of the parasite in the human host [16]. A blood schizonticide (chloroquine or any artemisinin-based combination therapy, except the combination with sulfadoxine-pyrimethamine) is given to kill the asexual blood stage rapidly to relieve the patient of acute signs and symptoms. A second drug, an 8-aminoquinoline, is given either daily (for primaquine) over 14 days or in a single dose (for tafenoquine) to kill the hypnozoites lying dormant in the hepatocytes and prevent relapse [16, 20–22]. The anti-hypnozoite therapy with either primaquine or tafenoquine is effective, safe, and relatively well-tolerated in a compliant patient with a normal G6PD activity. However, due to the major drawback of 8-aminoquinolines, they are contraindicated in individuals with G6PD deficiency [16].

Mauritania is the only country in West Africa where *P. vivax* has become highly endemic in recent years [23]. Since 2013, the Mauritanian Ministry of Health recommends the systematic use of primaquine, in addition to artesunate-amodiaquine or artemether-lumefantrine, to treat all cases of *P. vivax* malaria, in line with the World Health Organization (WHO) recommendations [16, 24, 25]. However, to date, the new drug policy has remained a dead letter, not only because primaquine (or tafenoquine) is not available in the country, but also because health care workers do not have access to information about the G6PD status of individual patients, which requires the measurement of G6PD enzyme activity using ultraviolet (UV) spectrophotometry as the gold standard method [26–29].

To overcome this technical difficulty in the field, several point-of-care devices have been developed in recent years for a rapid screening procedure [30–32]. These diagnostic devices characterize the G6PD phenotype of an individual by measuring enzyme activity and can be classified into qualitative and quantitative tests. The qualitative tests are designed to discriminate G6PD-deficient individuals from those with intermediate and normal enzyme activity, usually at the cut-off threshold of 30% of normal activity. They are particularly useful to screen G6PD deficiency in hemizygous males and homozygous females with two mutant alleles associated with G6PD deficiency [30, 31]. The design, items contained in the test packet, test cassette (or test strip), and procedures of qualitative tests for G6PD screening are similar to those of existing rapid diagnostic test (RDT) for malaria, which will facilitate the training of health personnel. A quantitative test may be more sensitive than qualitative tests to identify G6PD-deficient individuals and determine the level of activity in heterozygous females if its values are highly correlated with those of spectrophotometry. Although the results of both quantitative and qualitative tests can be obtained within 15 min, the principal disadvantages of a quantitative test are the higher cost and requirement for a specific instrument [30]. The present study was conducted with the aim to evaluate two rapid diagnostic test systems in Nouakchott: the qualitative CareStart™ RDT and the quantitative CareStart™ G6PD biosensor. Due to various financial and technical difficulties in obtaining a spectrophotometer and training a laboratory technician to use the equipment properly, G6PD genotyping was performed in the present study to serve as a gold standard against which the screening tools were measured.

## Methods

### Study sites and patients

Outpatients aged > 1 year old consulting spontaneously at Teyarett health centre, Sebkha health centre, or Maternity and Infant Hospital Centre (Centre Hospitalier mère-enfant) in Nouakchott were included in 2019–2020. The exclusion criteria were hospitalized patients, patients presenting at the emergency department, patients with severe disease, and patients with altered states of consciousness. After explaining the purpose of the study and obtaining an informed consent, basic information on age, sex, and ethnic origin was recorded in a survey questionnaire that was subsequently anonymised. Ethnic origin is a pertinent information in this study because Mauritania is a multi-ethnic country and ethnic origin is known to be associated with G6PD genotype [4]. No attempt was made in this study to include a representative and comparable sample of males and females of different ethnic origin.

Fingerprick capillary blood samples (approximately 200 µl) were collected to perform point-of-care rapid diagnostic tests for G6PD phenotype and determination of haemoglobin level. In addition, two or three drops of capillary blood (approximately 100–150 µl) were imbibed onto Whatman 3 mm filter paper, air dried, and stored at -20 °C for DNA analysis.

### Phenotyping

G6PD phenotype was determined using two field-applicable point-of-care tools: the qualitative RDT for G6PD (CareStart™ G6PD RDT; Access Bio, Inc., Somerset, NJ) and quantitative CareStart™ G6PD biosensor (Access Bio, Inc., Somerset, NJ). The RDT for G6PD was performed to screen G6PD deficiency according to the manufacturer’s instructions. This RDT is designed to detect < 30% of normal G6PD activity, but it is most suitable for detecting class I and II G6PD deficiency [26], which corresponds to severe G6PD deficiency with < 10% of normal activity, according to the manufacturer. The quantitative G6PD biosensor was used in parallel to measure enzyme activity. Approximately 5 µl of capillary blood was transferred to the strip of the device. The biosensor determines G6PD enzyme activity in 4 min and automatically adjusts the reading to the ambient temperature. In addition to qualitative and quantitative tests for G6PD, haemoglobin level was measured using a portable HemoCue device (HemoCue AB, Ängelholm, Sweden) to normalize G6PD enzyme activity for haemoglobin.

### Genotyping

Earlier studies have established that one of the most commonly observed G6PD genotypes in sub-Saharan Africa is the African-type G6PD A−, which is associated with a point mutation in nucleotide 376 and a second mutation in 202, 542, 680, or 968 [33–36]. In North Africa, as well as around the Mediterranean basin, the Mediterranean-type G6PD B−, which is associated with a single point mutation in nucleotide 563, is frequently found in people suffering from favism [37, 38]. Based on this information, nucleotide changes in 202, 376, 542, 563, 680, and 968 were investigated by polymerase chain reaction (PCR)-restriction fragment length polymorphism (RFLP) and/or sequencing in a stepwise manner in the present study.

In the first step, a fragment of exon 5 spanning nucleotide 376 and another fragment of exons 6–7 spanning nucleotide 563 were amplified separately by PCR using the protocol developed by Carter et al. [36] and Ezz El-Deen et al. [39]. All primer sequences were verified and corrected based on the complete G6PD sequence [2]. The fragments of exon 5 and exons 6–7 were digested by *Fok* I and *Mbo* II, respectively. The sample proceeded to the second step of the protocol if the mutant nucleotide 376G was found. PCR was performed to amplify a fragment of exon 4, which was digested with *Nla* III to determine nucleotide 202 (G6PD A^−(202)^) [36]. If the sample yielded 376G mutation in the first step and wild-type G202 in the second step, the third step consisted of PCR amplification of exon 9, followed by restriction digestion with *Nci* I [36]. If the wild-type T968 was found in the third step, exons 6–7 amplified in the initial step to determine nucleotide 563 were sequenced in the fourth step to determine nucleotides 542 and 680. Sequencing also allowed a second verification to exclude 563T (Mediterranean-type G6PD B−).

### Data and statistical analysis

G6PD enzyme activity measured by CareStart G6PD biosensor was expressed as International Units/dL (IU/dL). G6PD activity was normalized for haemoglobin values measured at the same time as G6PD measurement and expressed as IU/g haemoglobin (IU/g Hb). G6PD activity is influenced by haemoglobin level. Anaemia, in particular, can increase G6PD activity due to reticulocytosis or on the contrary decrease G6PD activity due to chronic blood loss [26]. The WHO classification of haemoglobin levels to diagnose anaemia was used to classify patients who were anaemic at the time of G6PD measurement [40]. Based on this classification, non-anaemia, mild anaemia, moderate anaemia, and severe anaemia in male and female children < 5 years old were defined as ≥ 11.0 g/dL, 10.0–10.9 g/dL, 7.0–9.9 g/dL, and < 7.0 g/dL, respectively. In male and female children aged between 5 and 11 years old, non-anaemia, mild anaemia, moderate anaemia, and severe anaemia corresponded to ≥ 11.5 g/dL, 11.0–11.4 g/dL, 8.0–10.9 g/dL, and < 8.0 g/dL, respectively. In male and female children who were 12–14 years of age, as well as non-pregnant women aged ≥ 15 years old, non-anaemia, mild anaemia, moderate anaemia, and severe anaemia were defined as ≥ 12.0 g/dL, 11.0–11.9 g/dL, 8.0–10.9 g/dL, and < 8.0 g/dL, respectively. For men aged ≥ 15 years old, non-anaemia, mild anaemia, moderate anaemia, and severe anaemia were defined as ≥ 13.0 g/dL, 11.0–12.9 g/dL, 8.0–10.9 g/dL, and < 8.0 g/dL, respectively.

The genotype of normal G6PD enzyme without any mutations in males and females is designated *Gd*^B^ or, more commonly these days, G6PD B and BB, respectively [26]. If there was only one mutation in nucleotide 376, without any of the 4 nucleotide changes assessed in the present study, the genotype was referred to as G6PD A in hemizygous male and AA or BA in females. These three genotypes with a single A376G nucleotide change are not associated with G6PD deficiency. A normal G6PD without deficiency therefore refers to one of the following: A or B (in males), and AA, BB, or BA (in females). The African-type G6PD A− was defined as the presence of two mutations, A376G and one of the following: 376G + 202A (referred to as G6PD A^−(202)^), 376G + 968C (called G6PD A^−(968)^ or Betica-Selma), 376G + 542T (called G6PD A− Santamaria), or 376G + 680T (G6PD A^−(680)^). The African-type genotype is designated A− in hemizygous male, A−A− in homozygous female, and AA− or BA− in heterozygous female. In the presence of 563T mutation (without the African-type G6PD mutations since they are mutually exclusive), the sample was classified as the Mediterranean-type G6PD B−.

Descriptive statistics and performance of the screening tests were calculated using GraphPad Prism (GraphPad software; San Diego, CA). Qualitative variables were compared using Fisher’s exact test. The level of significance of all statistical tests was set at *P* < 0.05.

## Results

### Characteristics of patients

**Fig. 1:**
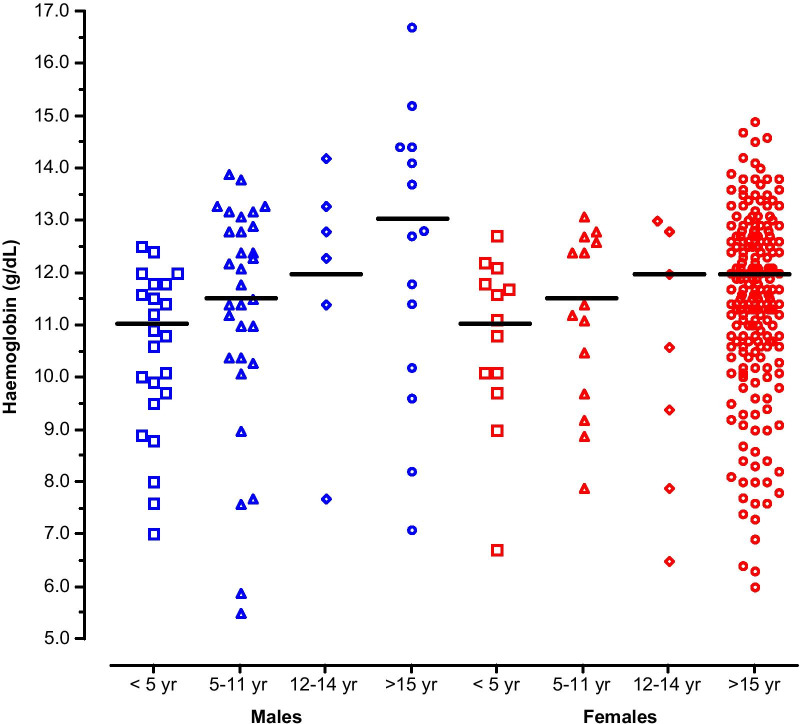
Distribution of individual haemoglobin values of Mauritanian patients included in the study according to sex (males, blue; females, red) and age groups (years, yr) used in the WHO classification [40]. The horizontal bars denote cut-offs above which the patient is considered non-anaemic

A total of 323 patients (74 males and 249 females) were included in the present study (Table 1). The sex ratio (M:F, 0.30) was highly skewed towards adult women because most patients (*n* = 255) were recruited at the Maternity and Infant Hospital Centre. A total of 68 patients were recruited at the other health centres (23 at Teyarett health centre and 45 at Sebkha health centre). The overall mean age [± standard deviation (*SD*); range] of the patients was 23.6 ± 14.4 (1.1–91) years old. The mean age of males (11.4 years old) was significantly younger (*P* < 0.05) than that of females (27.2 years old). There were 142 (44.0%) white Moors, 74 (22.9%) black Moors, 67 (20.7%) Pulars, 23 (7.1%) Wolofs, 10 (3.1%) Soninkes, 1 (0.3%) black Moor-Pular, 1 (0.3%) black Moor-Wolof, 1 (0.3%) Pular-Soninke, and 4 (1.2%) patients of unknown ethnic origin (missing data). The mean haemoglobin (± *SD*; range) of the patients was 11.3 ± 1.8 g/dL (5.5–16.7 g/dL). A total of 37 (50.0%; 95% *CI*: 38.1–61.9%) males and 141 (56.6%; 95% *CI*: 50.2–62.9%) females were anaemic, based on sex- and age-adjusted cut-off thresholds established by the WHO (Table 1; Fig. 1) [40]. The majority of these patients (*n* = 145) had mild to moderate level of anaemia; 24 had severe anaemia, mostly near the borderline level.

**Table 1:** Characteristics of included patients

Characteristics	Males	Females
Number	74	249
Mean age (± *SD*; range) (years)	11.4 ± 14.6 (1.1–70)	27.2 ± 12.2 (1.1–91)
Age groups (years)* 1–18 (*n*, %) ≥ 18 (*n*, %)	62 (83.8) 12 (16.2)	44 (17.7) 20582.3)
Ethnic groups (*n*, %)** White Moors Black Moors Pular Wolof Soninke Black Moor-Fulani or Wolof Pular-Soninké	45 (60.8) 18 (24.3) 4 (5.4) 2 (2.7) 2 (2.7) 0 1 (1.4)	97 (39.0) 56 (22.5) 63 (25.3) 21 (8.4) 8 (3.2) 2 (0.8) 0
Mean haemoglobin (± *SD*; range) (g/dL)	11.3 ± 2.2 (5.5–16.7)	11.4 ± 1.7 (6.0–14.9)
Anaemia*** Children < 5 year (< 11.0 g/dL) (*n*/*N*, %) Children 5–11 year (< 11.5 g/dL) (*n*/*N*, %) Children 12–14 year (< 12.0 g/dL) (*n*/*N*, %) Adults > 15 year old (< 12.0–13.0 g/dL) (n/N, %)	13/23 (56.5) 14/31 (45.2) 2/6 (33.3) 8/14 (57.1)	7/13 (53.8) 8/14 (57.1) 4/7 (57.1) 122/215 (56.7)

*n* number of patients with the characteristics, *N* total number of patients in the group, *SD* standard deviation

*The age of majority is 18 years old in Mauritania

**White Moors are an ethnic group of Arab-Berber origin. Black Moors, Pular, Wolof, and Soninke are of black African ancestry. Four missing data on ethnic groups (2 among males and 2 among females)

***Haemoglobin levels and age groups were based on the World Health Organization (WHO) classification [40]. For adults, anaemia is defined as < 13.0 g/dL in men and < 12.0 g/dL in non-pregnant women. The cut-off for anaemia in pregnant women (< 11.0 g/dL) is lower than that of non-pregnant women. It was assumed that none of the included female patients were pregnant because information on possible pregnancy was not obtained and pregnancy test was not performed

### G6PD genotypes

Of 74 males, 69 had either the wild-type genotype B (*n* = 61) or genotype A (i.e., with a single A376G mutation; *n* = 8), both of which are associated with normal G6PD activity (Table 2). Five carried the African-type A- genotype: G6PD A^−(202)^ G202A/A376G (*n* = 3) or G6PD A^−(968)^ Betica-Selma A376G/T968C (*n* = 2) (Table 3). Among 5 males with the African-type A− genotype, 3 were black Moors (2 G6PD A^−(202)^ and 1 G6PD A^−(968)^), 1 was white Moor (G6PD A^−(202)^), and 1 was Soninke (G6PD A^−(968)^).

**Table 2:** G6PD genotypes of Mauritanian patients included in the study

Ethnic group	Males (*n*, %)				Females (*n*, %)						
	*N*	B	A	A-	*N*	BB	BA	AA	BA-	AA-	A-A-
White Moors	45	43 (95.6)	1 (2.2)	1 (2.2)	97	85 (87.6)	7 (7.2)	1 (1.0)	4 (4.1)	0	0
Black Moors	18	8 (44.4)	7 (38.9)	3 (16.7)	56	38 (67.9)	6 (10.7)	1 (1.8)	6 (10.7)	4 (7.1)	1 (1.8)
Pular	4	4 (100)	0	0	63	33 (52.4)	17 (27.0)	1 (1.6)	11 (17.5)	1 (1.6)	0
Wolof	2	2 (100)	0	0	21	13 (61.9)	7 (33.3)	0	1 (4.8)	0	0
Soninke	2	1 (50.0)	0	1 (50.0)	8	5 (62.5)	0	1 (12.5)	1 (12.5)	0	1 (12.5)
Soninke/Pular	1	1 (100)	0	0	0	0	0	0	0	0	0
BM/Pular	0	0	0	0	1	1 (100)	0	0	0	0	0
BM/Wolof	0	0	0	0	1	0	0	1 (100)	0	0	0
Unknown	2	2	0	0	2	2 (100)	0	0	0	0	0
Total	74	61	8	5	249	177	37	5	23	5	2

The number of individuals (*n*) with different glucose-6-phosphate dehydrogenase (G6PD) genotypes and the number of individuals (*N*) belonging to different ethnic groups. Normal hemizygous males, A or B; African-type G6PD genotypes associated with deficiency in hemizygous male, A−; normal females, BB, AA, and BA; heterozygous females, AA− and BA−; homozygous females carrying African-type genotypes associated with G6PD deficiency, A−A−. Percentages in parentheses were calculated in relation to the total number of males or females belonging to different ethnic groups (*n*/*N*). Soninke/Pular, BM/Pular, and BM/Wolof refer to individuals whose parents belong to two distinct ethnic groups. *BM* Black Moor

**Table 3:** Frequency of G6PD A− allelic variants in individuals with G6PD A− genotypes in Nouakchott

Ethnic group	G6PD A- allelic variants					
	Male (*n*/*N*, %)		Female (*n*/*N*, %)			
	Hemizygous		Heterozygous			Homozygous
	202A	968C	202G/A	542A/T	968 T/C	202A/A
White Moor	1/45 (2.2)	0	2/97 (2.1)	1/97 (1.0)	1/97 (1.0)	0
Black Moor	2/18 (11.1)	1/18 (5.6)	9/56 (16.1)	1/56 (1.8)	0	1/56 (1.8)
Pular	0	0	1/63 (1.6)	1/63 (1.6)	10/63 (15.9)	0
Wolof	0	0	1/21 (4.8)	0	0	0
Soninke	0	1/3 (33.3)	0	0	1/8 (12.5)	1/8 (12.5)
Total	3/74 (4.1)	2/74 (2.7)	13/249 (5.2)	3/249 (1.2)	12/249 (4.8)	2/249 (0.8)

Data are expressed as the number of affected individuals with glucose-6-phosphate dehydrogenase (G6PD) variants (*n*), the total number of male or female individuals belonging to one of the ethnic groups (*N*), and percentage in parentheses. The mutations A376G + G202A denote African-type G6PD A^−(202)^. The double mutations A376 + T968C lead to G6PD A^−(968)^ Betica-Selma. None of the patients had A376G + A542T (G6PD A^−^ Santamaria), A376G + G680T (G6PD A^−(680)^), or C563T mutation (Mediterranean-type G6PD deficiency)

Among females, 219 had genotypes associated with normal G6PD (177 BB, 37 BA, and 5 AA), 28 were heterozygotes (5 AA− and 23 BA−), and 2 were homozygotes (A−A−) (Table 2). Among heterozygotes, 13 carried G6PD A^−(202)^, 12 G6PD A^−(968)^ Betica-Selma, and 3 G6PD A^−(542)^ Santamaria variants (Table 3). Heterozygous females belonged to white Moors (*n* = 4) and Mauritanians of black African ancestry (black Moors, *n* = 10; Pular, *n* = 12; Wolof, *n* = 1; Soninke, *n* = 1). Both homozygous women (one black Moor and one Soninke) had the African-type A^−(202)^ (376G/202A). None of the patients of both sexes had the Mediterranean-type G6PD genotype.

### Screening tests

**Fig. 2:**
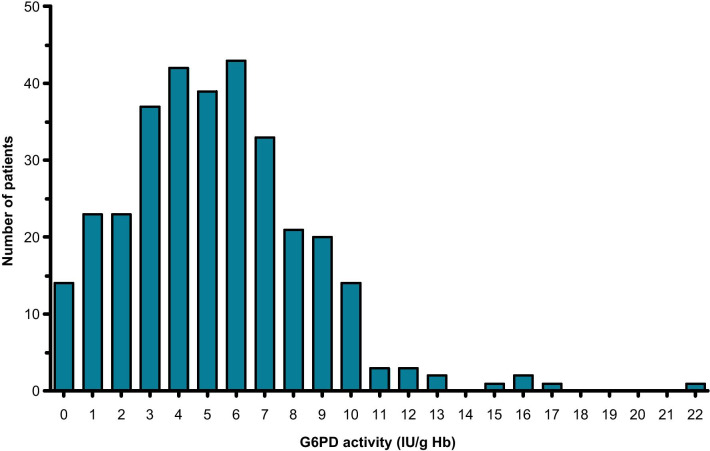
Glucose-6-phosphate dehydrogenase (G6PD) activity normalized for haemoglobin [International Unit/g haemoglobin (IU/g Hb)] was determined in Mauritanian patients (*n* = 323) by CareStart G6PD biosensor in Nouakchott

**Fig. 3:**
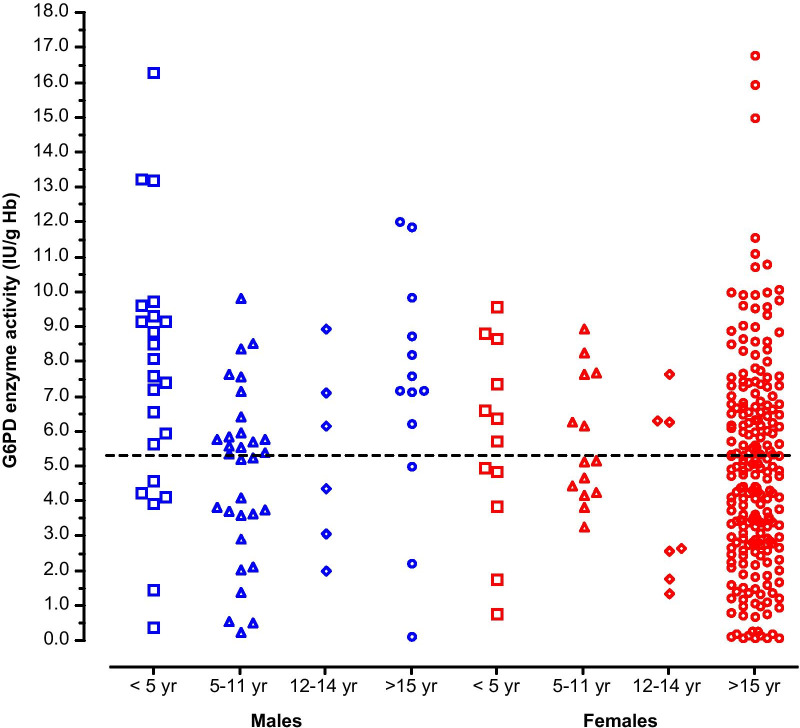
The dashed line denotes the median value of glucose-6-phosphate dehydrogenase (G6PD) activity found in male and female non-anaemic children aged > 1 year old (year) and adults (5.34 International Units/g haemoglobin [IU/g Hb]). One outlier datapoint (21.9 IU/g Hb) found in a girl aged less than 5 years is not presented in the graph. The age groups are those used in the World Health Organization (WHO) classification of haemoglobin levels to diagnose anaemia [40]

The distribution of G6PD activity determined by CareStart G6PD biosensor in Mauritanians included in the present study is illustrated in Fig. 2. Overall, the mean [± *SD*; 95% confidence intervals (95% *CI*)] activity was 5.35 ± 3.24 (4.94–5.70) IU/g Hb in 323 patients. The median (range) was 5.19 (0.08–21.94) IU/g Hb. The mean (± *SD*; 95% *CI*) and median (range) activities in different subsets of patient population were calculated (Table 4). The median value in the male population (*n* = 72) was 5.92 IU/g Hb. The adjusted median value in the male population, calculated by excluding those with genotypes associated with G6PD deficiency (i.e., hemizygous A−), was 6.18. The distribution of anaemia in different age groups in both sexes (Fig. 1) and the distribution of G6PD enzyme activity according to sex and age groups (Fig. 3) suggested that bias may have been introduced due to the presence of many patients with different levels of anaemia. To correct this bias, further calculations were made to reduce the effect of anaemia on G6PD activity (Table 4). In non-anaemic male population (*n* = 35) and male and female population (*n* = 130) with normal genotypes (i.e., A or B in males and AA, BB, or BA in females), the median G6PD activities were 5.72 and 5.34 IU/g Hb, respectively. Although the adjusted median of the male population (i.e., 6.18 U/g Hb) would have been used to set the 100% activity in a study using spectrophotometry as the reference method, the adjusted median of 5.34 IU/g Hb, determined from non-anaemic male and female population, was considered to correspond to 100% G6PD activity in the present study, in which genotyping was the reference method.

**Table 4:** Profile of G6PD activity in Mauritanian patients

Population	*N*	G6PD activity (IU/g Hb)			
		Mean (± *SD*)	95% *CI*	Median	Range
Male					
All male patients	72	6.10 ± 3.13	5.36–6.83	5.92	0.12–16.29
Adjusted values	67	6.51 ± 2.83	5.82–7.20	6.18	1.46–16.29
Adjusted values, non-anaemic	35	5.69 ± 2.35	4.88–6.50	5.72	2.03–9.60
Male + Female					
All patients	323	5.35 ± 3.24	4.99–5.70	5.19	0.08–21.94
Adjusted values	285	5.72 ± 3.03	5.37–6.08	5.52	0.55–21.94
Adjusted values, non-anaemic	130	5.46 ± 2.50	5.02–5.89	5.34	0.72–11.58

The “adjusted values” refer to the mean and median calculated from the patient population, excluding those with genotypes associated with glucose-6-phosphate dehydrogenase (G6PD) deficiency, i.e., hemizygous males with A− genotype, homozygous (A−A−) females, and heterozygous (AA− and BA−) females. The adjusted values in in non-anaemic individuals (excluding mild, moderate, and severe anaemia) are based on individuals without genotypes associated with G6PD deficiency. The definition of anaemia and different levels of anaemia were based on the WHO classification of haemoglobin levels [40]. *N* number of patients, International Units/g haemoglobin(IU/g Hb); 95% *CI* 95% Confidence interval, *SD* standard deviation

CareStart G6PD RDT was positive in 8 patients. Among seven patients with A− or A−A− genotype, all but one were correctly identified as G6PD deficient by RDT. Six of these patients had G6PD activity ranging from 0.12 to 1.43 IU/g Hb, as measured by biosensor. One homozygous female with A^−(202)^ genotype in whom RDT yielded a negative result had G6PD activity of 2.34 IU/g Hb.

### Performance of G6PD biosensor

The G6PD activities in three males with G6PD A^−(202)^ genotype were 0.29 IU/g Hb (Hb 10.3 g/dL), 0.59 IU/g Hb (Hb 10.1 g/dL), and 1.43 IU/g Hb (Hb 13.3 g/dL) and those in two males with G6PD A^−(968)^ Betica-Selma were 0.12 IU/g Hb (Hb 8.2 g/dL) and 0.38 IU/g Hb (Hb 8.0 g/dL). Based on 100% activity set at 5.34 IU/g Hb, these activities in males with A− genotypes corresponded to 2.2–26.8% of the normal activity. CareStart G6PD RDT was positive in all of these five male patients, indicating G6PD deficiency. Two homozygous females with A^−(202)^ genotype had G6PD activities of 2.34 IU/g Hb (Hb, 10.7 g/dL; RDT negative) and 0.14 IU/g Hb (Hb, 7.3 g/dL; RDT positive), corresponding to 43.8% and 2.6% of the normal enzyme activity, respectively.

The performance of G6PD biosensor is best assessed against spectrophotometric measurement of G6PD activity not only because the latter is the gold standard but also because they are both phenotypic tests. Nonetheless, in the present study, the performance of G6PD biosensor was assessed against genotyping (Table 5). AA− (*n* = 5) and BA− (*n* = 23) heterozygous females, but not BA heterozygotes, were excluded from analysis because it is known that their phenotype cannot be predicted from genotype. At 10% cut-off level, its sensitivity was low (57.1%), and the specificity was high (98.6%). At 30% cut-off, the sensitivity and specificity were 85.7% and 91.7%, respectively. At 80% cut-off, the sensitivity was 100% while the specificity was 64.9%. The sensitivity and the specificity did not improve when patients with severe anaemia (*n* = 24) were excluded from analysis. Further analysis in a subset of patients without mild to moderate anaemia was not performed because only one patient with the G6PD genotype associated with African-type G6PD deficiency was non-anaemic.

**Table 5:** Performance of CareStart G6PD biosensor against genotyping

Performance	Cut-off levels		
	< 10%	< 30%	< 80%
Cut-off value (IU/g Hb)	0.53	1.60	4.27
All patients except AA- and BA- females			
* n* (%)	8 (2.7)	30 (10.2)	108 (36.6)
Sensitivity (%; 95% *CI*)	57.1 (18.4–90.1)	85.7 (42.1–99.6)	100 (59.0–100)
Specificity (%; 95% *CI*)	98.6 (96.5–99.6)	91.7 (87.8–94.6)	64.9 (59.1–70.4)
Positive predictive value (%; 95% *CI*)	50.0 (23.8–76.2)	20.0 (13.3–28.9)	6.5(5.6–7.5)
Negative predictive value (%; 95% *CI*)	99.0 (97.6–99.6)	99.6 (97.7–99.9)	100
Patients without severe anaemia*			
* n* (%)	7 (2.6)	27 (9.9)	102 (37.4)
Sensitivity (%; 95% *CI*)	50.0 (11.8–88.2)	83.3 (35.9–99.6)	100 (54.1–100)
Specificity (%; 95% *CI*)	98.5 (96.2–99.6)	91.8 (87.8–94.8)	64.0 (58.0–69.8)
Positive predictive value (%; 95% *CI*)	42.9 (17.6–72.6)	18.5 (11.7–28.0)	5.9 (5.1–6.8)
Negative predictive value (%; 95% *CI*)	98.9 (97.5–99.5)	99.6 (97.6–99.9)	100

Data were analyzed in a total of 295 patients, including females characterized to be BA heterozygote, except for 5 AA− and 23 BA− heterozygous females, and a subset of patients without severe anaemia (n = 273), as defined by the World Health Organization (WHO) [40]. The number (*n*) of patients denotes those whose glucose-6-phosphate dehydrogenase (G6PD) activity was less than 10%, 30% or 80% of the normal G6PD activity determined by CareStart G6PD biosensor. 100% G6PD activity was defined as 5.34 International Units/g haemoglobin (IU/g Hb) in the present study, as shown in Table 4. *95% CI* 95% confidence interval

Assuming the reliability of G6PD biosensor measurement, the possibility to administer primaquine to patients on the basis of G6PD activity was simulated and assessed (note: the patients included in the present study were not malaria-infected). The results are summarized in Table 6. About one-fourth of males and females included in this study had at least 80% of G6PD activity, which was supported by genotyping showing one of the genotypes associated with normal G6PD activity. The presence of > 80% of G6PD activity is compatible with the safe administration of a standard dose of primaquine (0.25 mg base/kg body weight/day for 14 days) or tafenoquine (300 mg single dose) to kill hypnozoites [21, 22, 27]. About 15% and 3.4% of males and females of the study population had 30–80% and < 30% of residual enzyme activity, respectively. These patients would have been eligible for a modified primaquine treatment regimen, which consists of a weekly dose (0.75 mg base) for 8 weeks. Six of seven hemizygous and homozygous patients with the African-type genotype were anaemic, and four of these six anaemic patients had enzyme activity of 0.12–0.38 IU/g Hb, i.e., 2.2–7.1% of activity. The other two anaemic patients with African-type genotypes had 0.59 IU/g Hb (11.0% activity) and 2.34 IU/g Hb (43.8% activity). More than half of the patient population (54–57%) in the present study had varying degrees of anaemia, which may lead to an unreliable measurement of G6PD activity. In real situations, before primaquine treatment can be re-considered for these patients, quantitative G6PD testing will be required after anaemia is corrected.

**Table 6:** G6PD activity level and possibility to administer primaquine

G6PD activity level	Males (*n*, %)	Females (*n*, %)
< 30% (deficient)	1 (1.4)	10 (4.0)
30–80% (intermediate)	13 (17.6)	34 (13.7)
> 80% (normal activity)	20 (27.0)	65 (26.1)
Unreliable due to anaemia	40 (54.1)	140 (56.2)
Total	74 (100)	249 (100)

A standard dose of 8-aminoquinolines (primaquine or tafenoquine) can be administered in patients with normal glucose-6-phosphate dehydrogenase (G6PD) activity [16]. A weekly dose (0.75 mg base) of primaquine for eight weeks can alternatively be prescribed to patients with intermediate or deficient activity. 8-aminoquinolines are contraindicated in pregnant or breastfeeding women and infants < 6 months old [16]. The category “unreliable” level denotes the presence of anaemia which renders G6PD measurement unreliable. In anaemic patients, known causes of anaemia should first be corrected and G6PD activity re-evaluated after several weeks

## Discussion

As one of the first steps to facilitate implementation of the new drug policy including primaquine, the current prevalence of G6PD genotypes in Mauritania was assessed in our recent works [41, 42]. The preliminary database showed that, overall, 6.3% of Mauritanian men and 1.5% of Mauritanian women are affected by G6PD genetic polymorphisms leading to the African-type G6PD deficiency, and that the Mediterranean-type G6PD deficiency is absent [42]. Moreover, these studies have shown that G6PD deficiency tends to occur much more frequently in Mauritanian ethnic groups of Black African ancestry. The present study is the second phase of the preliminary efforts to control *P. vivax* malaria in the country. CareStart™ G6PD RDT and CareStart™ G6PD biosensor were assessed under real conditions in health centres and in a hospital in Nouakchott.

CareStart™ G6PD RDT performed poorly in the present study, contrary to the conclusion of systematic reviews and meta-analysis of earlier studies conducted elsewhere [28, 31]. Although the RDT was positive, indicating < 30% of G6PD activity, in six of seven patients with African-type genotype associated with G6PD deficiency, numerous false negatives were observed. One possible explanation may be poor storage condition and/or high ambient temperature during screening. The recommended storage condition of the kit is ambient temperature, and the optimal temperature for its use is between 18 and 32 °C. The ambient temperature can exceed 35 °C in Nouakchott during certain months of the year. Another drawback of this qualitative screening test is the difficulty of interpretation when there is a slight colour change. The difference between normal G6PD activity (distinct colour change to violet) and deficiency (slight colour change to violet or no colour change) may be difficult to distinguish, especially because there is no control band that indicates that the test is valid or not. Further improvements are needed to conserve the quality of RDT for G6PD in a hot, dry climate, as in most of Mauritania where *P. vivax* occurs.

By contrast, CareStart™ G6PD biosensor is a user-friendly point-of-care device with automatic temperature adjustment that performed better than CareStart™ G6PD RDT when compared to G6PD genotyping at the cut-off level of 80%. The first-generation G6PD biosensor was used in the present study. Other earlier studies also reported that the performance of CareStart™ G6PD biosensor needs further improvement. In a study conducted by Weppelmann et al. in Haiti [43], the sensitivity and the specificity of biosensor, compared to spectrophotometry, were 53.7% and 94.6% at < 60% G6PD activity and 5.9% and 99.7% at < 30% activity, respectively. Alam et al. [44] found the sensitivity and the specificity of biosensor, compared to spectrophotometry, of 71% and 98% at < 70% enzyme activity, the level beyond which tafenoquine can be administered, in Bangladesh. The optimal cut-off to obtain 91% sensitivity and 82% specificity at < 70% enzyme activity was 6.8 IU/g Hb after adjustment of 100% activity. The sensitivity and the specificity of the second-generation biosensor, compared to spectrophotometry, were similar, i.e., 79% and 98%, respectively, at 70% cut off in another study performed in Thai-Burmese border only after the initial threshold for biosensor was corrected using Youden’s index [45]. In our study, the adjustment of median G6PD values using receiver operating characteristic (ROC) curve analysis and Youden’s index to ameliorate the sensitivity and the specificity of G6PD biosensor was not attempted as in other studies because spectrophotometry was not used to validate the measurements of biosensor [44–46].

The results of G6PD biosensor in our study generally support those of other authors conducted in different epidemiological context [43–46]. In practice, however, there are several caveats associated with the use of this device. First, haemoglobin measurement is required in parallel with G6PD activity. The first-generation CareStart G6PD biosensor used in the present study does not have a measurement of haemoglobin incorporated in the device, necessitating a separate device to determine the haemoglobin level. In patients who are anaemic at the time of G6PD testing, the result of G6PD activity may be either normal or high due to reticulocytosis or low due to blood loss. In either case, G6PD activity during the anaemic phase does not reflect the baseline G6PD activity of the individual. If known, the cause(s) of anemia should be treated first, and G6PD testing repeated after several weeks. The drawback is that there is currently no clear guideline on whether to or how to administer anti-hypnozoite drug in such circumstances. Second, before primaquine is administered to non-anaemic women, a precise measurement of G6PD enzyme activity with a reliable quantitative assay is required, as qualitative screening tests cannot reliably detect G6PD deficiency in heterozygous women. In this context, the enzymatic activity measured by G6PD biosensor may have to be calibrated against spectrophotometry for each patient population in different countries. Until present, spectrophotometric measurement of G6PD activity has not been performed in Mauritania. G6PD genotyping may circumvent this problem, but it is inconceivable to extend this practice in the field. Third, there is no universally accepted normal range for G6PD activity. Each country is required to determine the normal G6PD activity of its population measured with a quantitative gold standard procedure. The cut-off value calculated in the present study cannot be extrapolated to other methods or to populations who were excluded, in particular new-borns and infants less than 1 year old. Fourth, even if anaemic patients are excluded from immediate treatment with primaquine, other haematological conditions, such as haemoglobinopathies and leukocytaemia, may yield a normal G6PD test result in a G6PD-deficient patient [6].

One of the limitations of the present study is the lack of comparison of the performance of biosensor with that of the gold standard, spectrophotometry. Although other authors have confirmed the high reliability of biosensor, which gave results that are highly correlated with those of spectrophotometry [43–47], high correlation does not imply that biosensor yields the same enzyme activity as spectrophotometry. In the study conducted by Alam et al. [44], the correlation of enzyme activity measurements between biosensor and spectrophotometry was 0.773, and discordant results were obtained in some patient samples at < 30% enzyme level. Bancone et al. also reported that CareStart G6PD biosensor overestimates enzyme activity compared to spectrophotometry [45]. As a pilot study to assess the feasibility and practicality of CareStart G6PD biosensor, the present study was not designed to determine 100% G6PD activity and cut-off levels. The value of 100% G6PD activity based on the adjusted median found in the present study should therefore be considered as a preliminary estimate in Mauritania. Future work plan before implementing *P. vivax* elimination programme should include a comparison of G6PD activity measured by biosensor and spectrophotometry in a large sample of general population representing different ethnic backgrounds with an aim to define the normal values in the Mauritanian population. Another limitation of this study is the overrepresentation of females in our study population. For studies on an X-linked disorder, such as G6PD deficiency, studies in males would yield a higher number of subjects with the disorder to facilitate the calculation of cut-off threshold of G6PD deficiency while limiting the major confounding factor, i.e., heterozygosity associated with unpredictable G6PD activity in females due to the phenomenon called lyonisation [48–50]. However, other studies on the assessment of G6PD biosensor have also included more females than males, as in the works of Alam et al. (73% females) and Bancone et al. (72% females) [44, 45]. Genotyping is at present the only method that allows an accurate identification of heterozygous females. More investigations on G6PD involving females are required to resolve the problem of primaquine treatment in heterozygous females. Moreover, an updated version of WHO classification of G6PD variants and recommendation for 8-aminoquinoline treatment is needed for implementation of *P. vivax* control programmes in Mauritania and elsewhere in the world [51].

## Conclusions

The present study is the first of its kind in Mauritania that assessed the utility of G6PD biosensor. Because of the predominance of *P. vivax* malaria in Nouakchott and elsewhere in northern part of the country, a point-of-care G6PD testing is of primordial importance in ensuring that primaquine is administered to *P. vivax*-infected patients with normal G6PD activity and that treatment leads to complete cure. Further work with G6PD biosensor, in parallel with spectrophotometric measurement, will be required before full deployment of this point-of-care tool in the field in Mauritania.

## Data Availability

All data generated and analysed during this study are included in this published article.

## Abbreviations

G6PD: Glucose-6-phosphate dehydrogenase
NADPH: Nicotinamide adenine dinucleotide phosphate hydrogen
PCR: Polymerase chain reaction
RDT: Rapid diagnostic test
ROC: Receiver operating characteristic
RFLP: Restriction fragment length polymorphism
UV: Ultraviolet
WHO: World Health Organization
